# Role of habitual diet in metabolic fuel utilization and metabolic flexibility, evidence in Kenyan and U.S. cohorts

**DOI:** 10.1038/s41430-025-01665-3

**Published:** 2025-09-19

**Authors:** Pablo Torres-Aguilar, Anna M. R. Hayes, Clay Swackhamer, Emmanuel Ayua, Laura Michelin, Violet Mugalavai, Bruce R. Hamaker

**Affiliations:** 1 Department of Food Science, Nelson Hall of Food Science, Whistler Center for Carbohydrate Research, Lafayette, IN USA; 2 Department of Food Science and Human Nutrition, University of Illinois Urbana Champaign, Urbana, IL USA; 3 Department of Food Science and Nutrition, University of Eldoret, Eldoret, Kenya

**Keywords:** Risk factors, Metabolic diseases

## Abstract

**Background/objectives:**

Animal studies support that diet affects metabolic fuel utilization and metabolic flexibility. We hypothesized that individuals with contrasting dietary patterns would have different metabolic responses. Differences in metabolic fuel utilization, metabolic flexibility, and gastric emptying time to carbohydrate challenges (rapidly vs slowly digestible carbohydrates [RDC/SDC]) were assessed between US and Kenyan cohorts consuming diets characteristic of each population.

**Subjects/methods:**

We assessed metabolic fuel utilization using a portable breath CO_2_ measuring device and gastric emptying in two cohorts (Kenya, *n* = 23; US, *n* = 13) for 2 h following RDC and SDC challenges. Study meals, matched in energy content (732 kJ), consisted of test carbohydrates (30 g) mixed into applesauce (200 g). An estimated respiratory exchange ratio (RER_est_) was calculated from the CO_2_ values. Metabolic flexibility (MF) was assessed using Percent Relative Cumulative Frequency followed by modeling with the Weibull Cumulative Distribution function. We collected dietary data using three 24-h dietary recalls and used multivariate mixed effect models to assess dietary influences on RER_est_/MF to carbohydrate challenges.

**Results:**

Kenyan participants had higher RER_est_ and greater MF compared to US participants regardless of the carbohydrate challenge (*P* < 0.0001), and had improved MF response with SDC vs RDC. Multivariate Model 1 (macronutrient composition) showed that carbohydrate (*P* = 0.02) and protein (*P* < 0.001) were predictive of RER_est_; and for Model 2 (carbohydrate quality), total fiber (*P* = 0.026), starch (*P* = 0.001) and added sugars (*P* < 0.001) were predictive of RER_est_.

**Conclusion:**

The Kenyan cohort consuming a diet of high carbohydrate quality and low in fat showed greater carbohydrate oxidation and improved MF.

## Introduction

Metabolic flexibility (MF), defined as the body’s ability to efficiently switch between energy sources (e.g., carbohydrates and fats) depending on availability or need [1, 2], is closely linked to metabolic health [1, 3]. Lack of MF is associated with chronic conditions like obesity and type 2 diabetes [3–6]. Proposed interventions to improve MF, including caloric restriction and strenuous exercise [3, 7, 8], are unsustainable for long periods of time [9, 10]. Recent animal studies suggest diet influences MF [11–14]; however, human evidence remains limited [15, 16]. We recently showed that consumption of slowly digestible carbohydrates (SDCs) improves MF (i.e., switching between use of carbohydrate vs fat) and shifts metabolic fuel utilization toward fat oxidation in a mouse model [14]. However, there have only been a few studies comparing habitual or intervention diets on MF [15–17]. Here, we looked at metabolic fuel utilization, MF, and gastric emptying time in humans relative to differences in habitual diet and a rapidly digestible and slowly digestible carbohydrate challenge. We compared US and Kenyan cohorts with the hypothesis that Kenyans consuming a traditional diet high in SDCs (e.g., starchy thick porridge, *ugali*) may have better MF than US individuals consuming a diet high in RDC; and that they may utilize a SDC challenge differently.

This cross-sectional study compared MF between US and Kenyan cohorts while retrospectively analyzing their contrasting habitual diets. Kenyans consumed a traditional East African diet high in SDCs, fiber, and low in fat, while the US cohort followed a relatively poor-quality diet of Healthy Eating Index (HEI) ≤ 65. We used a portable, recently validated breath CO_2_ measuring device to estimate the respiratory exchange ratio (RER_est_) [18] in response to acute dietary carbohydrate challenges and assessed diet quality (HEI) using 24 h diet recalls. HEI is a tool that scores from 0–100 to measure how well an individual’s diet aligns with the 2015–2020 Dietary Guidelines for Americans [19]. A multivariate model tested the predictive power of dietary factors on RER_est_.

## Methods

### Study design

**Fig. 1:**
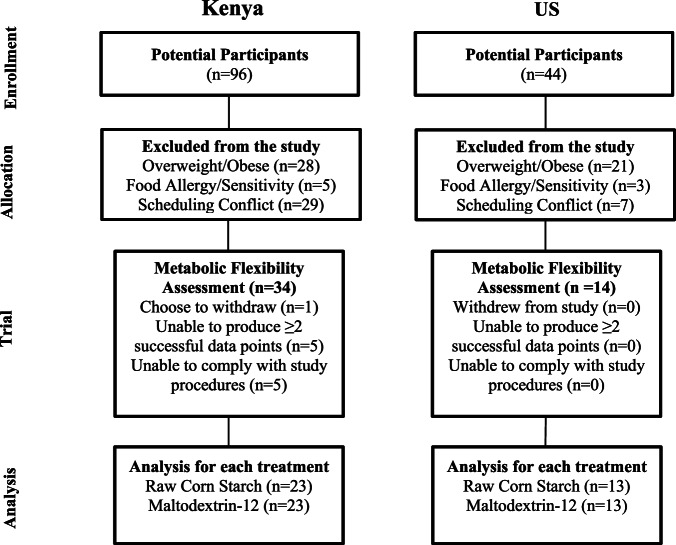
Participant recruitment and participation flow diagram. The diagram summarizes screening, exclusions, enrolment, and analysis in Kenya and the United States. In Kenya, 44 individuals were screened, 34 completed the metabolic flexibility training and 23 were analyzed per treatment arm. In the US, 96 individuals were screened, 14 completed the assessment and 13 were analyzed per treatment arm.

We conducted a two-center, crossover human trial at Purdue University (West Lafayette, IN, US) and University of Eldoret (Eldoret, Rift Valley, Kenya). Eligibility criteria included age between 18 and 50 years, normal BMI (18.5–25 kg/m^2^), and stable weight for the past 3 months. Kenyan participants needed to regularly consume a typical Kenyan diet, including SDC-rich *ugali* and traditional leafy greens ≥3 times/week, while US participants required a HEI ≤ 65. Exclusion criteria included history of gastrointestinal disease, diabetes, food allergies, medication use, smoking, pregnancy or nursing (Fig. 1).

Selected participants (*n* = 36) were randomized to determine the order in which they received the two test meals: one made with rapidly digestible carbohydrate (RDC) and one made with slowly digestible carbohydrate (SDC). There was a seven-day washout period between the different test meals. Participants fasted for 10 h overnight before tests. Participants avoided intense physical activity and alcohol consumption the day before test days. Participants were not adapted to a standardized diet.

Statistical power analysis was performed using an enrollment factor 2:1. Power for an unbalanced group, RER *µ*_1_ = 0.92, *µ*_2_ = 0.87, STD = 0.05. We needed 12 participants for the US cohort, and 24 participants for the Kenyan cohort for a one tail analysis with *σ* = 0.05 and *β* = 0.8.

### Ethics approval and consent to participate

All study procedures were conducted in accordance with relevant guidelines and regulations. Ethics approval was obtained from the Institutional Review Board of Purdue University (Protocol IRB-2019-206) and the Institutional Research and Ethics Committee at Moi University in Kenya (IREC protocol 3323) before enrollment. Informed consent was obtained from all participants prior to data collection. The study was registered at clinicaltrials.gov (ID NCT03630263).

### Test meals

Carbohydrate challenge test meals were semi-thick pastes of RDC or SDC powder mixed with unsweetened applesauce (~732 kJ total). The RDC test meal included 30 g (~447 kJ) of maltodextrin DE-12 (Dry MD 01909, Cargill, Minneapolis, MN, US) and the SDC meal included 30 g (~447 kJ) of raw corn starch (Argo, Memphis, TN, US), each mixed in 200 g (~285 kJ) of unsweetened applesauce (Musselman’s, Peach Glen, PA, US) with 0.2 g of xanthan gum (Bob’s Red Mill, Milwaukee, OR, US) to equalize viscosity (1200 Cp). Test meals were served at ambient temperature. Metabolic fuel utilization and gastric emptying time were assessed on different days.

### Estimated respiratory exchange ratio (RER_est_)

In this study, a portable device (Lumen®, Metaflow Ltd, Tel Aviv-Yafo, Israel) was used to measure breath CO_2_, as an indicator of metabolic fuel utilization (i.e., fat versus carbohydrate). The Lumen device utilizes a CO_2_ sensor and a flow sensor to measure the volume and concentration of exhaled CO_2_. The CO_2_ percentage is assessed through a standardized breathing maneuver that includes a 10 s breath hold, designed to maximize CO_2_ detection. O_2_ concentration is considered a fixed parameter, based on the physiological principle that O_2_ consumption is relatively constant under resting conditions [20].

Lumen® was validated against indirect calorimetry and has been used in other clinical studies [18, 21, 22]. While respiratory exchange ratio (RER) is the ratio of VCO_2_/VO_2_ in breath as a proxy to carbohydrate/lipid oxidation at the cellular level, Lumen’s calculations (based on %CO_2_) remain closer to dimensionless respiratory quotient [CO_2_production/O_2_consumption]. Lorenz et al (2021) used ordinary least squares regression to estimate RER measures from Lumen % CO_2_ values, with the assumption that RER is an accurate independent measure. A significant model effect was present (F1,63 = 18.54, *P* < 0.001 *R*^2^ = 0.2274). A 5.3665-unit increase in Lumen % CO_2_ was expected to increase RER_est_ by 1.00-unit, as shown (Eq. 1):

1 $${{RER}}_{{est}}=\frac{{{CO}}_{2}+0.7445}{6.111}$$

Although use of RER_est_ rather than RER is a limitation, its use in the present study facilitated access to an underserved population in Kenya.

### Assessment of metabolic flexibility

We estimated MF using the slope (*b*) and *x*_50_ parameters of the Weibull Cumulative distribution after expressing the RER_est_ values as percent relative cumulative frequency (PRCF). A higher slope (*b*) indicates reduced MF, while a high *x*_50_ (~1 on the x-axis) indicates greater carbohydrate utilization due to the collection of breath samples in the postprandial period after an overnight fast and carbohydrate-rich meal. To calculate parameters, we pooled RER_est_ data for all participants, calculated PRCF using the method proposed by Riachi et al. [23], and modelled it using the Weibull Cumulative Distribution Function [24]. Following calculation of PRCF, we fitted plots of RER_est_ (ascending order) vs. PRCF to the Weibull Cumulative Distribution function (Eq. 2).

2 $$y=1-{\text{exp}}\left(-{\left[\frac{x}{{x}_{50}}\right]}^{b}ln\left(2\right)\right)$$

Where:

y = percent relative cumulative frequency (PRCF; 0 to 100%);

*x*_50_ = median respiratory exchange ratio;

*b* = distribution breadth constant (dimensionless), indicative of slope.

We modelled using the “fitnlm” function with the nonlinear least squares method option in MATLAB (R2020a, The MathWorks, Inc., Natick, MA, US). Bounds were placed to ensure that *x*_50_ fell within the range of RER_est_ values for each dataset. An iterative modeling approach was used to obtain the best fit for each parameter, incorporating 5 initial “best guesses” to fit into the modeling approach. Modeling on pooled data was performed due to limitations (>20 data points needed) when using the Weibull Cumulative Distribution function [25, 26].

### Procedures during study visit

Before the study started, participants practiced and mastered the Lumen® breathing technique under staff supervision. Participants were allowed to rest/sit for 15 min before the baseline sample collection. After successful baseline sampling (fasting state), participants consumed the assigned test meal with water (100 mL) within 15 min. We collected post-meal breath samples for assessment of RER_est_ every 10 min for the first hour, and every 15 min for the second hour. Participants did not consume any other food or drink during the sessions.

### Diet assessment

Three 24 h dietary recalls were collected (one weekend day, two weekdays) using a pre-structured interview. Participants were provided with measuring cups for assessment of portion sizes. We analyzed dietary data using Nutrition Data System for Research software (NDSR) (Nutrition Coordination Center, Minneapolis, MN, US) [27]. Detailed recipes for Kenyan-specific food items were created, developed with input from a Kenyan women’s focus group (*n* = 6). Dietary data were normalized by dividing dietary intake by each participant’s body weight (e.g. kJ/kg BW; nutrient g/kg BW). Caloric and macronutrient intake (carbohydrate, protein, fat, and fiber), carbohydrate intake (fiber type, starch, added sugars, glycemic index), and diet quality (HEI) were calculated using NDSR software for both cohorts.

### Gastric emptying time

Assessment of gastric half-emptying time using the ^13^C octanoic acid breath test has been conducted in our laboratory for populations in the US and Africa [28–31]. For each test meal, 100 mg of ^13^C-octanoic acid (Sigma-Aldrich, Saint Louis, MO, USA) was mixed immediately before serving. Breath samples were collected at baseline (fasting) and every 15 min intervals for 2 h, then every 30 min from 2 to 4 h (Aluminized bags, Cambridge Isotope Laboratories, Tewksbury, MA, USA) and analyzed using infrared spectroscopy (POCone, Otsuka Electronics Co., Ltd., Osaka, Japan). The calculation of gastric emptying parameters is detailed elsewhere [31].

### Statistical analyses

We conducted statistical analysis using SAS version 9.4 (SAS Institute, Cary, NC, US). Multivariate, mixed effects models were used to assess the relationship between diet variables and RER_est_:

*Macronutrient composition | Model 1:* caloric intake, carbohydrate, protein, fat, including interactions between diet variables and interactions between diet variables and location.

*Carbohydrate quality | Model 2:* soluble fiber, insoluble fiber, starch, added sugars, including interactions between diet variables and interactions between diet variables and location.

*Diet quality | Model 3:* HEI and interactions between HEI and location.

During modeling, several diet variables showed multicollinearity. After evaluation, we modified the original proposed models to avoid collinear variables and only included variables with variance inflation factor <5. We removed the interactions between diet*diet and diet*location variables that exhibited multicollinearity. In model 1, caloric content (kJ/kg BW) and fat displayed multicollinearity and were removed. In model 2, soluble and insoluble fiber (g/kg BW) also displayed multicollinearity and were substituted for total fiber.

Metabolic parameters (CO_2_ and RER_est_) were analyzed using repeated measures two-way ANOVA (PROC MIXED) with test meal, location, diet variables, and time as fixed effects and subject as a random effect. Baseline values were added as covariates for all repeated measures analyses [32]. Homoscedasticity and normality of residuals were assessed using histograms and quantile-quantile plots. Data were normally distributed and did not require transformation. Significance level was set at *P* < 0.05, and Tukey’s post hoc test was conducted for multiple comparisons when the overall model was significant. Gastric emptying parameters (gastric half-emptying time, gastric lag phase, and gastric emptying coefficient) were analyzed using two-way ANOVA models (PROC MIXED) specifying test meal, location, and diet variables as a fixed effects and participant as a random effect.

For the Weibull modeling, we pooled all participant data from each group (test meal and cohort); therefore, there were no replicates. Confidence intervals (95%) were calculated for Weibull parameters (i.e. *x*_50,_ *b*) [33, 34]. Statistically significant differences (*P* < 0.05) were indicated by non-overlapping confidence intervals.

## Results

### Participant characteristics

Clinical characteristics of Kenyan (*n* = 23, 11 female/12 male) and US cohorts (*n* = 13; 9 female/4 male) are summarized as follows: The mean ± SD age was 32.9 ± 9.2 years for the Kenyan cohort and 23.9 ± 7.2 years for the US cohort. For height, the Kenyan cohort averaged 167.7 ± 9.1 cm, and the US cohort averaged 166.2 ± 9.9 cm. Average weight was similar (Kenyan: 66.6 ± 8.0 kg; US: 65.2 ± 11.1 kg), as was BMI (Kenyan: 23.6 ± 1.8; US: 23.5 ± 2.8).

### Diet assessment

Table 1 highlights habitual dietary differences between US and Kenyan cohorts. The US cohort consumed significantly more total calories, protein, fat, glucose, and maltose, but less total fiber, insoluble fiber, lactose, and added sugars compared to the Kenyan cohort. Both groups had similar carbohydrate, soluble fiber, starch, fructose, galactose, and sucrose intake. HEI scores were significantly higher for the Kenyan group (68.08) compared to the US group (48.89).

**Table 1:** Diet characteristics for the subgroups that underwent assessment of RERest.

	US *n* = 14					Kenya *n* = 23				
Variable	Mean	SD	% of total energy	Mean	SD	Mean	SD	Mean	SD	% of total energy
	(g/day)			(g/kg bw day)		(g/day)		(g/kg bw day)		
Carbohydrates	201.37	43.99	43.0	3.14^a^	0.71	226.88	46.36	3.50^a^	0.93	57.0
Protein	80.89	19.43	17.3	1.25^a^	0.26	54.65	18.72	0.84^b^	0.28	14.0
Fat	82.70	20.71	39.7	1.28^a^	0.27	50.34	18.66	0.77^b^	0.29	29.0
Total fiber	16.35	3.51		0.26^b^	0.06	21.66	6.93	0.33^a^	0.1	
Soluble fiber	5.29	1.27		0.08^a^	0.02	4.62	1.47	0.07^a^	0.02	
Insoluble fiber	10.99	2.45		0.17^b^	0.05	17.05	5.94	0.26^a^	0.08	
Starch	107.28	25.49		1.69^a^	0.51	125.12	29.07	1.93^a^	0.53	
Fructose	12.46	7.34		0.19^a^	0.1	9.96	6.76	0.15^a^	0.11	
Galactose	0.25	0.38		0.00^a^	0.01	0.08	0.27	0.00^a^	0	
Glucose	14.18	7.43		0.22^a^	0.08	8.95	5.97	0.13^b^	0.09	
Lactose	5.85	3.72		0.09^b^	0.05	20.27	14.07	0.33^a^	0.24	
Maltose	2.73	1.00		0.04^a^	0.01	0.93	1.01	0.01^b^	0.01	
Sucrose	26.36	20.84		0.40^a^	0.28	27.08	12.57	0.41^a^	0.19	
Added Sugars	55.54	35.51		0.83^b^	0.45	87.92	9.57	1.34^a^	0.17	
Caloric content (Kcal*/day)	1872.73	373.22		29.04^a^	5.38	1560.91	399.84	23.82^b^	6.94	
HEI				48.89^b^	9.1			68.08^a^	9.62	

*SD* standard deviation, *BW* body weight, *HEI* Healthy Eating Index. Superscript letters within rows indicate values significantly different by *t*-test, *p* < 0.05.

*0.239 kcal = 1 kJ.

### CO_2_, estimated respiratory exchange ratio (RER_est_), metabolic flexibility (MF), and gastric emptying

**Fig. 2:**
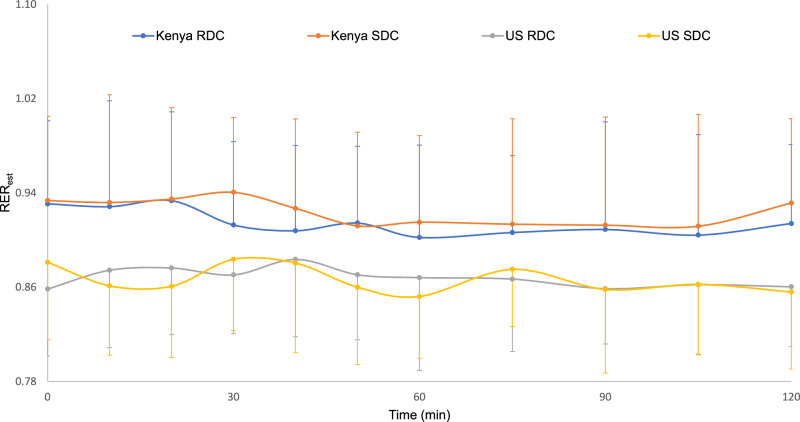
Assessment of RER_est_values over a 2 h period after consumption of either SDC or RDC meals. The X-axis shows time points in min (0, 10, 20, 30, 40, 50, 60, 75, 90, 105 and 120) and the Y-axis shows RER_est_ values. The curves indicate RER_est_ changes over time for SDC meal (30 g Raw Corn Starch + 200 g applesauce) or RDC meal (30 g maltodextrin DE-1 + 200 g applesauce). SDC—Kenya (Orange), RDC—Kenya (Blue), SDC—US (Yellow), RDC—US (Grey). Error bars represent ± standard deviation.

RER_est_ values were significantly higher in the Kenyan cohort compared to the US cohort (*P* < 0.0001; Fig. 2). Neither time (*P* = 0.40), test meal (*P* = 0.51), nor their interaction (*P* = 0.58) significantly influenced RER_est_. Thus, when presented with a carbohydrate challenge, the Kenyan cohort preferentially used more carbohydrate as a metabolic fuel source compared to the US cohort. RER_est_ response did not differ by RDC or SDC challenge, or during the postprandial period. There were no significant differences in gastric emptying parameters (Supplementary materials, Fig. 1).

**Fig. 3:**
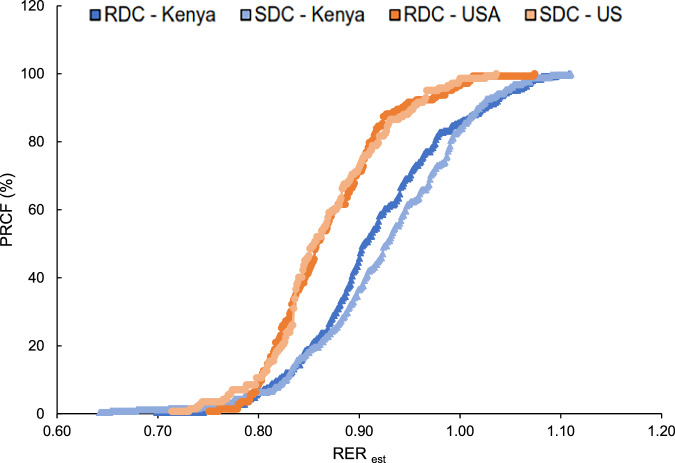
Percent relative cumulative frequency (PRCF) of pooled respiratory exchange ratio (RER _est_) values following SDC or RDC meal consumption in Kenyan and US cohorts. RER_est_ were calculated from CO2 values. The X-axis shows RER_est_ and the Y-axis shows PRCF (%). Data are pooled across participants (US *n* = 13, Kenya *n* = 23). Comparisons are between meals containing SDC (raw corn starch) or RDC (maltodextrin DE-12) in cohorts as follow, US RDC (dark orange); US SDC (light orange); Kenya RDC (dark blue); Kenya SDC (light blue). Because this is pooled data, there are no error bars.

We assessed MF using a Weibull Cumulative Distribution analysis to analyze the distribution of RER_est_ values in finer detail. In this approach, the slope (*b*) indicates the distribution of values: with a steeper slope (larger *b*) indicating values are concentrated in a small range (reduced MF), and a less steep slope (smaller *b*) indicating a wider distribution (greater MF). PRCF RER_est_ curves showed a broader spread and less steep slope for the Kenyan cohort than those from the US, indicating higher MF (Fig. 3). The slope (*b*) did not differ by carbohydrate challenge (RDC vs. SDC) across cohort locations.

**Fig. 4:**
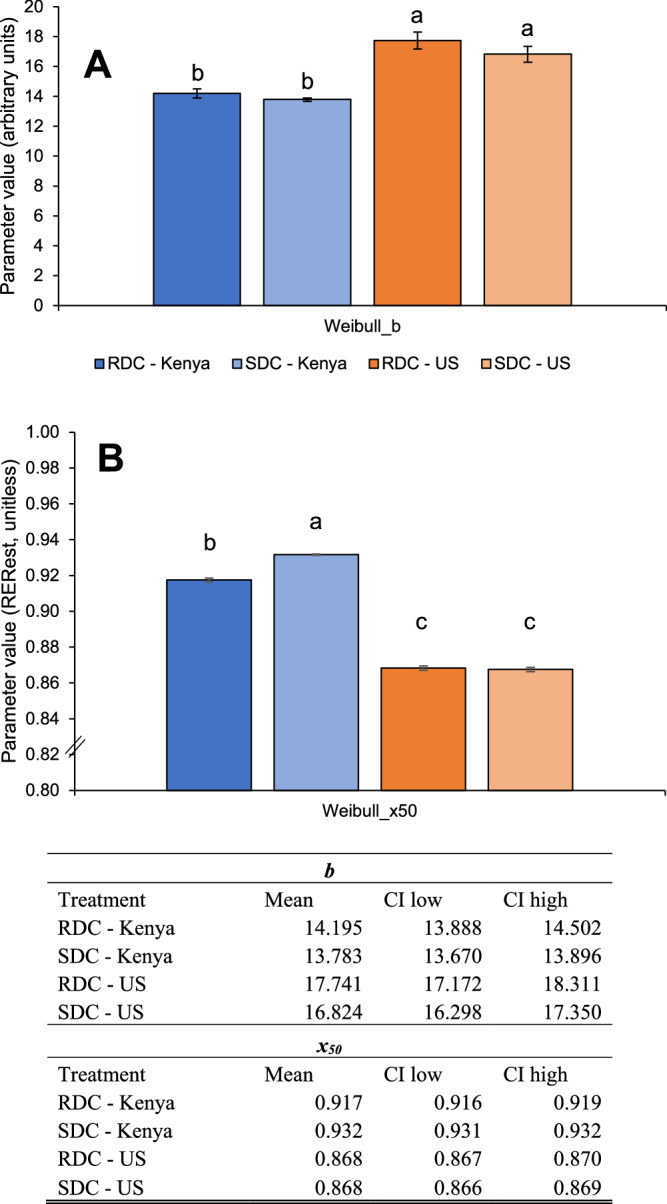
Weibull Cumulative Distribution parameters of pooled RER_est_ values following consumption of SDC or RDC. The X-axis displays treatment groups and the Y-axis shows Weibull function parameter values. Panel **A** indicates *b* as the distribution breadth constant or slope (dimensionless). Panel **B** displays *x*_50_ as the median RER_est_ during the postprandial period. Because this data is specifically depicting carbohydrate oxidation, a higher *x*_50_ may signify a more complete switch to carbohydrate oxidation (vs. fat oxidation), which is a hallmark of superior metabolic flexibility. Furthermore, a lower *b* indicates a broader spread in values, which suggests enhanced metabolic flexibility when specifically examining RER_est_ values. Different letters indicate statistically significant differences in parameter estimates per group (no overlap in 95% confidence intervals). US RDC (dark orange); US SDC (light orange); Kenya RDC (dark blue); Kenya SDC (light blue).

The second Weibull distribution parameter, *x*_50_, indicates the median metabolic fuel substrate used by the population. The Kenyan cohort had a higher *x*_50_ than the US cohort, regardless of the carbohydrate challenge (Fig. 4). Among Kenyans, the SDC challenge resulted in higher *x*_50_ (i.e., median value for carbohydrate oxidation) compared to the RDC challenge, suggesting a more complete ‘switch’ to carbohydrate oxidation following consumption of a carbohydrate-rich challenge meal containing slowly digestible starch (Figs. 3, 4). We did not observe differences between RDC and SDC consumption in the US group. Similar results were obtained with Lumen raw CO_2_ values (Supplementary materials, Fig. 2).

In summary, Kenyan participants had higher *x*_50_ (increased median RER_est_, indicative of carbohydrate oxidation) and lower *b* (broader range in RER_est_ values), showing increased MF compared to the US cohort. This was more pronounced for the SDC challenge in the Kenyan cohort, indicating greater carbohydrate oxidation.

### Predictive power of diet variables on RER_est_

Multivariate models showed significant associations between dietary factors and RER_est_ (Table 2). The predictive RER_est_ equations (Eqs. 3–8) for the three multivariate models are:

**Table 2:** Multivariable associations between RERest response and diet variables.

*Model 1 Macronutrient composition*				
Effect	Coefficient	95% CI		*P* value
		Lower limit	Upper limit	
(Intercept)	1.00	0.97	1.03	**<0.0001**
Location (US vs Kenya)	−0.03	−0.05	–0.01	**<0.001**
Carbohydrate (g/kg BW)	−0.01	−0.02	0	**0.02**
Protein (g/kg BW)	−0.05	−0.08	−0.03	**<0.001**
CL = 95% confidence intervals				
*Model 2 Carbohydrate quality*				
Effect	Coefficient	Lower limit	Upper limit	*P* value
(Intercept)	1.03	0.99	1.07	**<0.0001**
Location (US vs Kenya)	–0.08	–0.1	−0.06	**<0.0001**
Total fiber (g/kg BW)	0.12	0.01	0.22	**0.026**
Starch (g/kg BW)	–0.04	−0.05	−0.02	**0.0001**
Added sugars (g/kg BW)	–0.06	–0.08	−0.04	**<0.0001**
CL = 95% confidence intervals				
*Model 3 Diet quality*				
Effect	Coefficient	Lower limit	Upper limit	*P* value
Intercept	0.826	0.781	0.872	**<0.0001**
Treatment (maltodextrin vs raw corn starch)	–0.006	−0.013	0.001	0.094
Location (US vs Kenya)	−0.024	–0.043	–0.006	**0.01**
HEI	0.001	0.001	0.002	**<0.0001**

Bolded *P* values are statistically significant after correction for multiple comparisons using Tukey mean separation.

*CL* 95% confidence intervals, *HEI* healthy eating index, *BW* body weight.

*Model 1 macronutrient composition*

3 $${\text{For}}\,{\text{Kenya}}\,{{RER}}_{{est}}=+1.00+0.01{x}_{1}+0.05{x}_{2}$$

4 $${\text{For}}\,{\text{US}}\,{{RER}}_{{est}}=+0.97+0.01{x}_{1}+0.05{x}_{2}$$

Where *x*_1_= carbohydrate; *x*_2_=protein (g/kg of body weight)

*Model 2 carbohydrate quality*

5 $${\text{For}}\,{\text{Kenya}}\,{{RER}}_{{est}}=+1.03+0.12{x}_{1}-0.04{x}_{2}-0.06{x}_{3}$$

6 $${\text{For}}\,{\text{US}}\,{{RER}}_{{est}}=+0.95+0.12{x}_{1}-0.04{x}_{2}-0.06{x}_{3}$$

Where *x*_1_= total fiber; *x*_2_=starch; *x*_*3*_=added sugars (g/kg of body weight)

*Model 3* Diet quality

7 $${\text{For}}\,{\text{Kenya}}\,{{RER}}_{{est}}=+0.83+0.001{x}_{1}$$

8 $${\text{For}}\,{\text{US}}\,{{RER}}_{{est}}=+0.80+0.001{x}_{1}$$

Where *x*_1_ = HEI value

These models were used to assess the factors influencing RER_est_ (ranging from 0.7 to 1). Table 2 shows the direction and impact of each dietary component. In Model 1 (macronutrients), protein (*P* < 0.001, coeff = −0.05) had a stronger influence on RER_est_ than carbohydrates (*P* = 0.02). In Model 2 (carbohydrate quality), total fiber (*P* = 0.026, coeff = 0.12) had the greatest impact, while starch (*P* = 0.0001, coeff = −0.04) and added sugars (*P* < 0.001, coeff = −0.06) had smaller effects. In Model 3 (diet quality), HEI significantly influenced RER_est_ but had only a minor impact.

## Discussion

In this study, we compared the effect of habitual dietary differences between a Kenyan and an American cohort on RER_est_ and MF responses, and to RDC and SDC challenges. While we hypothesized that diet alone would be the factor driving these differences, we found that location also contributed to RER_est_ (i.e., intercepts of equations were somewhat different).

The Kenyan cohort only included sub-Saharan Africans, while the US cohort was mixed race (6 Caucasian, 2 Latino, 3 Asian, 2 African American). Sub-Saharan Africans have been shown to have higher RQ values compared to Caucasian populations, even after controlling for age, sex, physical activity, and lean body mass [35]. Additionally, while age and sex distributions differed modestly between cohorts, prior evidence suggests that metabolic flexibility is primarily driven by diet composition [1, 3]. In this study, the Kenyan cohort started with a higher baseline RER_est_ compared to the US cohort. Following carbohydrate challenges, we observed that the US cohort switched less effectively to carbohydrate as a metabolic fuel source (RER_est_ ~1), indicative of metabolic inflexibility. This observation was independent of digestibility of the carbohydrate challenge (Fig. 2). The metabolic inflexibility observed in the US cohort is supported by the percent relative cumulative frequency (PRCF) data and Weibull Cumulative Distribution analysis. PRCF plots with Weibull analysis provide a method to visualize the cumulative distribution of RER_est_ values, offering insight into both the central tendency (*x*_50_) and variability (slope) of metabolic responses. The US cohort displayed *x*_50_ values favoring fat oxidation (median SDC 0.85, median RDC 0.85) compared to the Kenyan cohort favoring carbohydrate oxidation (median SDC 0.93, median RDC 0.90) after the carbohydrate challenges (Figs. 3, 4). This is supported by narrower RER_est_ value distributions in the US cohort PRCF plots compared to the Kenyan cohort; a steeper slope (larger *b*) indicates RER_est_ values clustered in a small range, suggesting limited variability and therefore reduced MF. This contrasts with the Kenyan cohort that displayed a more gradual slope (smaller *b*), reflective of a broader spread of RER_est_ values, indicative of greater variability in substrate utilization and hence greater metabolic flexibility.

In our analysis, postprandial-to-fasting measurements were collected at a ratio of 10:1, with RER_est_ values recorded at 10, 20, 30, 40, 50, 60, 75, 90, and 120 min postprandial time (fasting value recorded and coded as 0 min). This high-resolution time series robustly captures dynamic metabolic adaptations through greater variability in substrate utilization across the postprandial period. Prior evidence indicates insulin and glucose reach peak concentrations approximately 45 min postprandially [32]. Follow-up PRCF analysis (Supplementary materials, Figs. 3–6) using datapoints from fasting to 50 min postprandial time showed the same results as for the 0–120 min full postprandial period, showing that these findings were robust with respect to the length of time in the postprandial period that was chosen for analysis.

We hypothesized that the Kenyan cohort, consuming a traditional diet rich in SDCs, would exhibit greater MF compared to the US cohort [14] and respond differently to an SDC challenge. Our findings support that the Kenyan cohort primarily oxidized carbohydrates rather than fats for energy and that they were more metabolically flexible compared to the US cohort. This was similarly shown by Fernandez-Calleja et al. (2018), who showed increased carbohydrate oxidation in mice after consumption of “lowly-digestible” starch [11]. The *x*_*50*_ value for the Kenyan cohort after consuming the SDC challenge was significantly greater than that of the RDC meal (0.932 vs 0.917, *p* < 0.05), whereas in the US cohort, there was not a significant difference in the *x*_*50*_ values between the SDC and RDC meals. This suggests that an MF-enhancing effect of SDC was only present in the Kenyan cohort, possibly revealing the effect of the habitual diet of Kenya to promote a physiological adaptation for favorable response to SDC consumption. To date, this is the first study evaluating carbohydrate digestibility and MF in healthy individuals.

The intrinsic habitual dietary characteristics significantly contributed to the observed metabolic responses, particularly carbohydrate and dietary fiber consumption. The US cohort followed a Western dietary pattern, high in protein and fat. High-fat diets have been associated with lower MF [16]. The Kenyan cohort consumed a lower fat diet, containing SDCs (as the thick maize porridge, *ugali*, was consumed >3 times per week), compared to the US cohort. Maize, sorghum, and millet *ugali* are traditional low glycemic foods consumed ubiquitously in East Africa beginning in early childhood [36–41]. Continuous consumption of SDCs in the Kenyan cohort might contribute to the improved MF. While there are no studies on humans linking carbohydrate digestibility and MF, animal studies support this hypothesis [11, 14]. Fernandez Calleja and collaborators [11] showed that continuous, early-life consumption of SDC (Amylogel ™, 50–75% native high amylose starch) in female mice increased life-long carbohydrate oxidation and MF after carbohydrate challenges.

We hypothesized that habitual diet would significantly impact RER_est_ and MF. While acute dietary changes have shown an effect on metabolic fuel utilization [41–44], the effect of habitual diet on metabolic flexibility remains understudied. We tested the effect of dietary pattern variables on RER_est_ using a multivariate model, aiming to reveal whether there could be long-term influences of diet on MF in healthy adults

Model 1 showed that carbohydrate and protein intakes increase RER_est_, with protein having a greater impact (coefficient 0.05). In this study, the US cohort consumed less carbohydrates (Table 2), although these were highly digestible. The Kenyan cohort consumed a diet containing SDCs supported by daily consumption of *ugali* (6-7 times/week, data not shown). The observed MF response in the US cohort might relate to the high consumption of RDCs, since high-fat and high-sucrose diets impair muscle mitochondrial function and decreased MF in rats exposed to the diets for 12 weeks [45]. Similar findings have been found in overweight men after consumption of high-fat diet for 3 weeks, with the control group (low-fat diet) showing a slight but significant increase in RER (+0.02) while in the high-fat diet group RER decreased (-0.05) [16]. Although model 1 focused on carbohydrate and protein, an alternate model with only fat rendered the same predictive power (coefficient -0.07); however, this model didn’t account for differences in cohort location.

For Model 2 (carbohydrate quality), total fiber, starch, and added sugars were independently correlated to RER_est_, with total fiber having the greatest impact (+0.12). There is limited evidence on the influence of dietary fiber on MF; however, its positive effect observed in our study might relate to a combination of reduced energy absorption in the intestinal tract [46] and the by-products of colonic fermentation activating pathways for glucose [47] and lipid metabolism [48] at the cellular level.

For Model 3, we found minimal effect of HEI on RER_est_. This is supported by a recent six-week study comparing a healthy diet (high in fruits and vegetables, pulses, fibers, nuts, fatty fish, and low in high-glycemic carbohydrates) vs. Western diet (25% energy mono/disaccharides +35% energy from fat) in obese and overweight individuals (50-70 years of age) finding that diet quality didn’t improve MF, whole body insulin sensitivity, or glycemic control [15]. It is important to note that the above study was performed in obese/overweight individuals in which MF might already be compromised, while the current study was done on lean individuals.

This study is among the first to explore the relationship between habitual diet and metabolic flexibility in free-living, healthy individuals across two nutritionally distinct populations. The inclusion of two demographically distinct cohorts from Kenya and the US allowed for a cross-sectional comparison, capturing the metabolic consequences of traditional versus Westernized diets in free-living individuals with similar BMI. The use of a within-subjects, crossover design minimized inter-individual variability and allowed for direct comparison of metabolic responses to carbohydrates with varying degrees of digestibility. Additionally, we employed a novel modeling approach using percent relative cumulative frequency and Weibull cumulative distribution parameters to assess metabolic flexibility, offering a more nuanced analysis of RER_est_ distribution than traditional mean-based methods. The implementation of a portable breath analysis technology (Lumen) enabled assessment of metabolic function in an understudied population, supporting the feasibility of conducting metabolic research in developing countries.

Our study also presents several limitations. We used estimated respiratory exchange ratio (RER_est_) derived from CO₂ measurements obtained via a portable breath analyzer, which does not account for oxygen consumption (VO₂). While validated against indirect calorimetry under resting conditions, RER_est_ may be less accurate in capturing true substrate oxidation dynamics, and its use limits the physiological precision of our estimates of MF. Also, participants followed their usual diet prior to testing, and dietary intake was not standardized in the days leading up to the test meals. While this design choice aligns with the aim to assess habitual dietary influences, it may introduce variability in pre-test metabolic states. Due to methodological constraints, the Weibull cumulative distribution modeling was performed on pooled data, precluding individual-level replication and limiting the granularity of our interpretation. Finally, we did not include direct clinical biomarkers of metabolic health (e.g., insulin sensitivity, fasting glucose), which limits our ability to link metabolic flexibility to broader metabolic outcomes.

## Conclusion

Cohorts in the US and Kenya, with similar BMI, had distinct responses in metabolic fuel utilization to habitual diets. The Kenyan cohort had higher carbohydrate oxidation and improved MF that was associated with consumption of a healthy diet comparably low in fat and high in SDCs. Furthermore, the Kenyan cohort showed greater MF following the SDC versus the RDC carbohydrate challenge. Our findings substantiate that habitual dietary characteristics significantly impact metabolic response leading to better MF, and, in this new finding, that the dietary patterns of the traditional Kenyan diet are associated with a marker of good metabolic health.

## Supplementary information

Supplementary material

## Data Availability

The datasets generated during and/or analyzed during the current study are available from the corresponding author on reasonable request.
