# Supplementary material for: Role of habitual diet in metabolic fuel utilization and metabolic flexibility, evidence in Kenyan and U.S. cohorts
Source: Eur J Clin Nutr. 2025 Sep 19;80(1):121–9. doi: 10.1038/s41430-025-01665-3 (PMC12783047; doi:10.1038/s41430-025-01665-3)
Supplement: Supplementary file 1 — Supplementary material [file 41430_2025_1665_MOESM1_ESM.pdf]

Supplementary materials

Figure 1

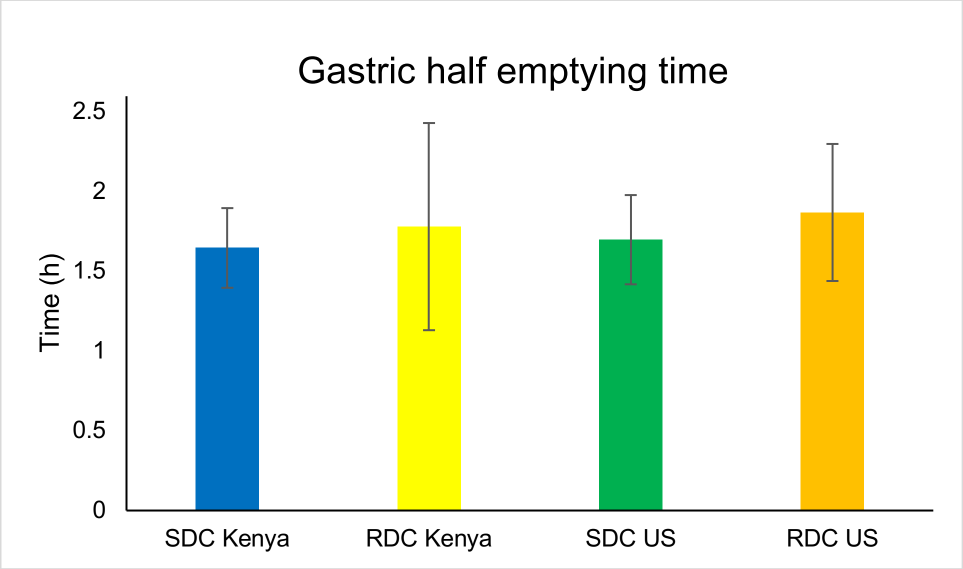

Mean half-gastric emptying values for Kenya and US after consumption of either slowly digestible carbohydrate meal (30 g RCS + 200 g applesauce) or rapidly digestible starch meal (30 g MDX + 200 g applesauce).

Figure 2

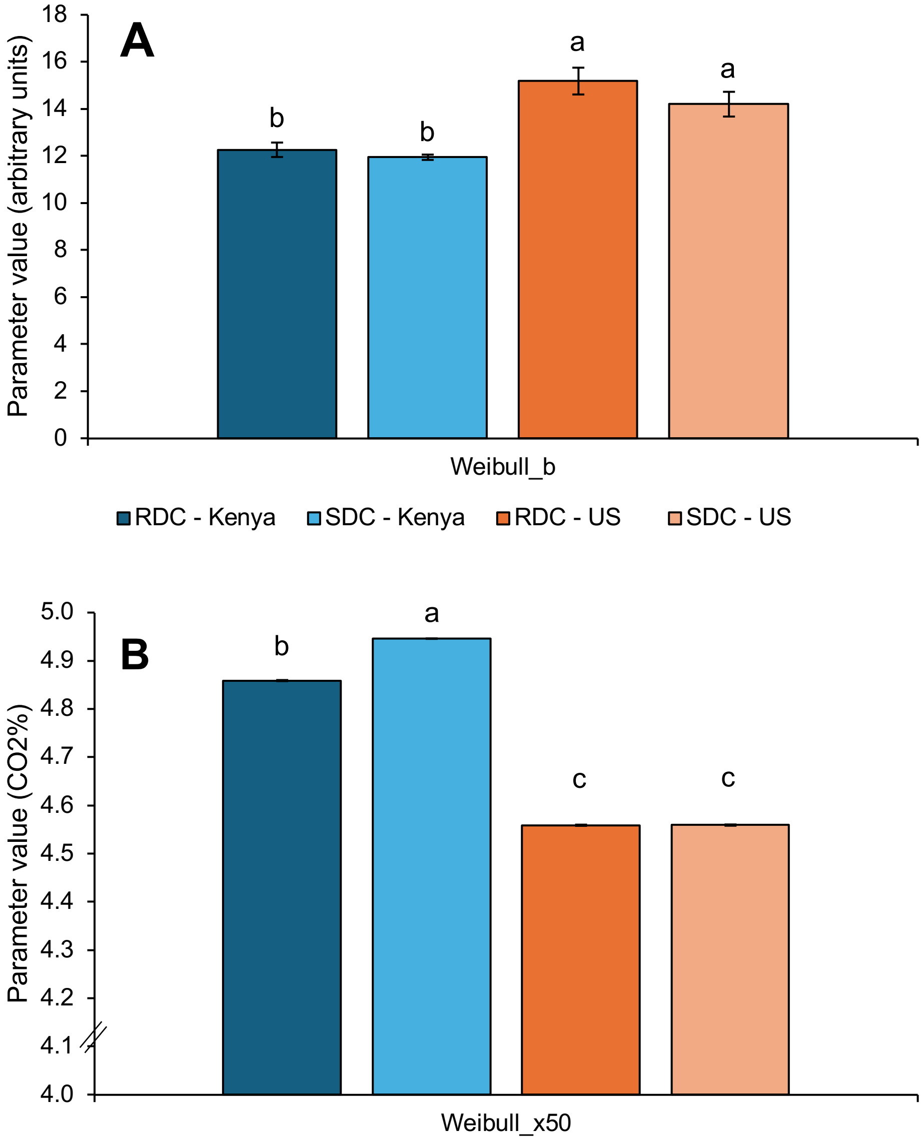

Weibull Cumulative Distribution parameter estimates from modeling percent relative cumulative frequency (PRCF) of pooled CO_2 values among all participants (US n=13, Kenya n=23) following consumption of SDC (raw corn starch) or RDC (maltodextrin DE-12). x_{50} represents the median CO_2 during the postprandial testing period, whereas b signifies the distribution breadth constant or slope (dimensionless). Because this data is specifically depicting carbohydrate oxidation, a higher x_{50} may signify a more complete switch to carbohydrate oxidation (vs. fat oxidation), which is a hallmark of superior metabolic flexibility. Furthermore, a lower b indicates a broader spread in values, which suggests enhanced metabolic flexibility when specifically examining CO_2 values. Different letters indicate statistically significant differences in parameter estimates per group (no overlap in 95% confidence intervals). US RDC (dark orange); US SDC (light orange); Kenya RDC (dark blue); Kenya SDC (light blue).

Figure 3

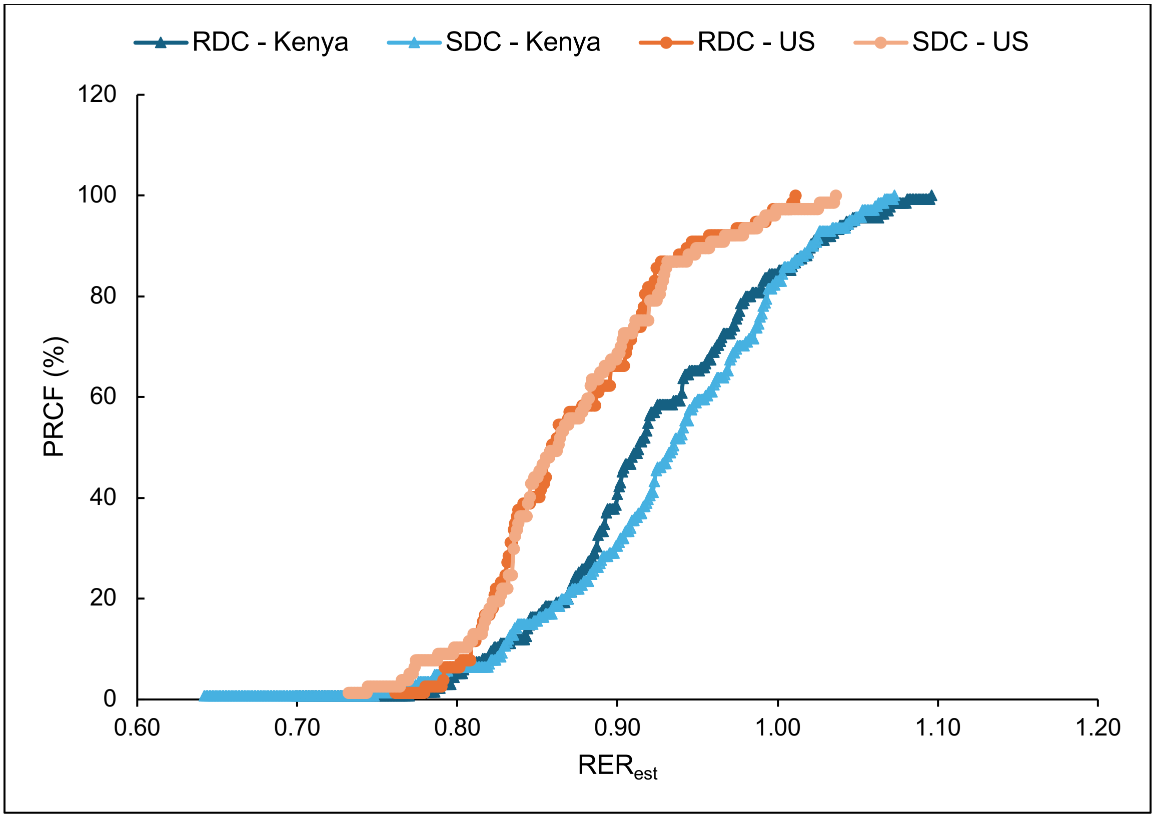

Percent Relative Cumulative Frequency (PRCF) analysis for RER_{est} values from **0-50 min.**

Figure 4

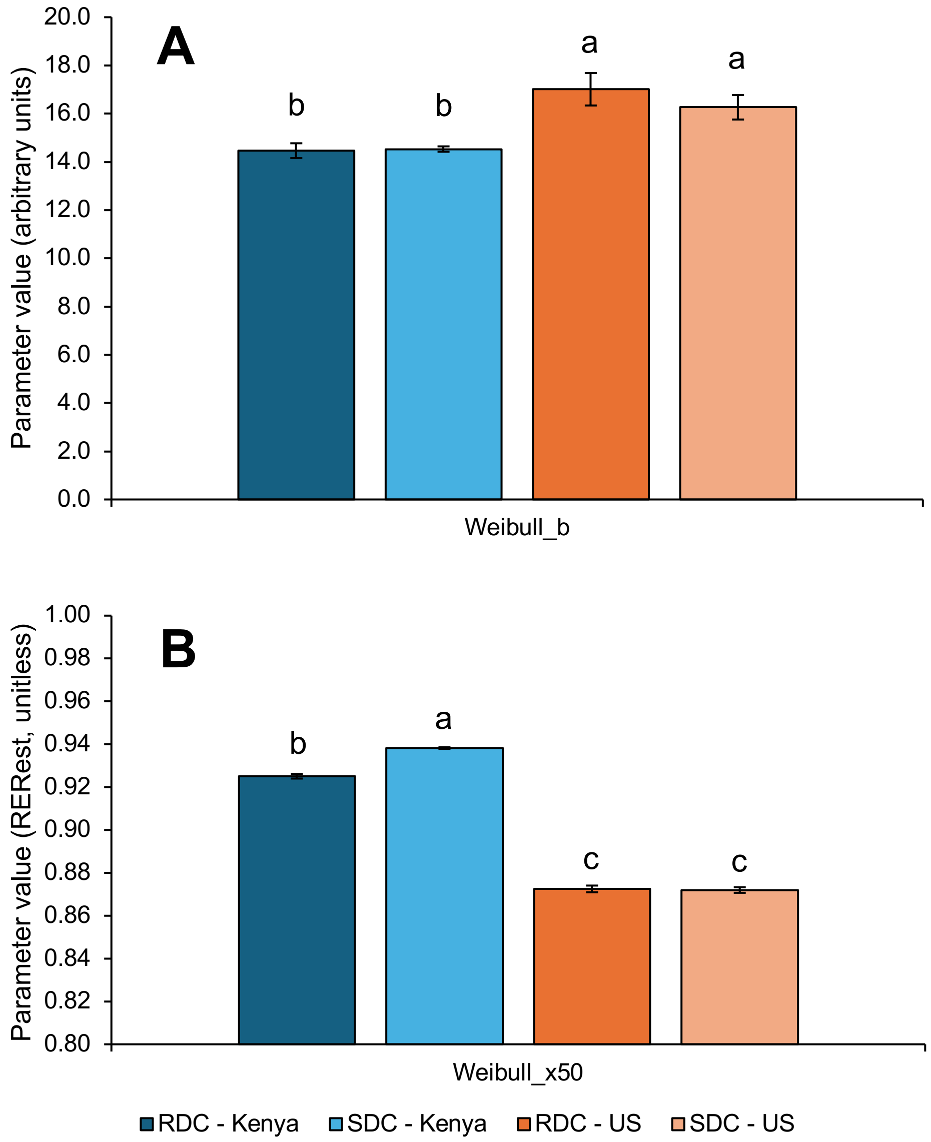

Weibull fit parameter estimates for RER_{est} data from **0-50 min**. Error bars represent ±95% confidence intervals (CI).

Figure 5

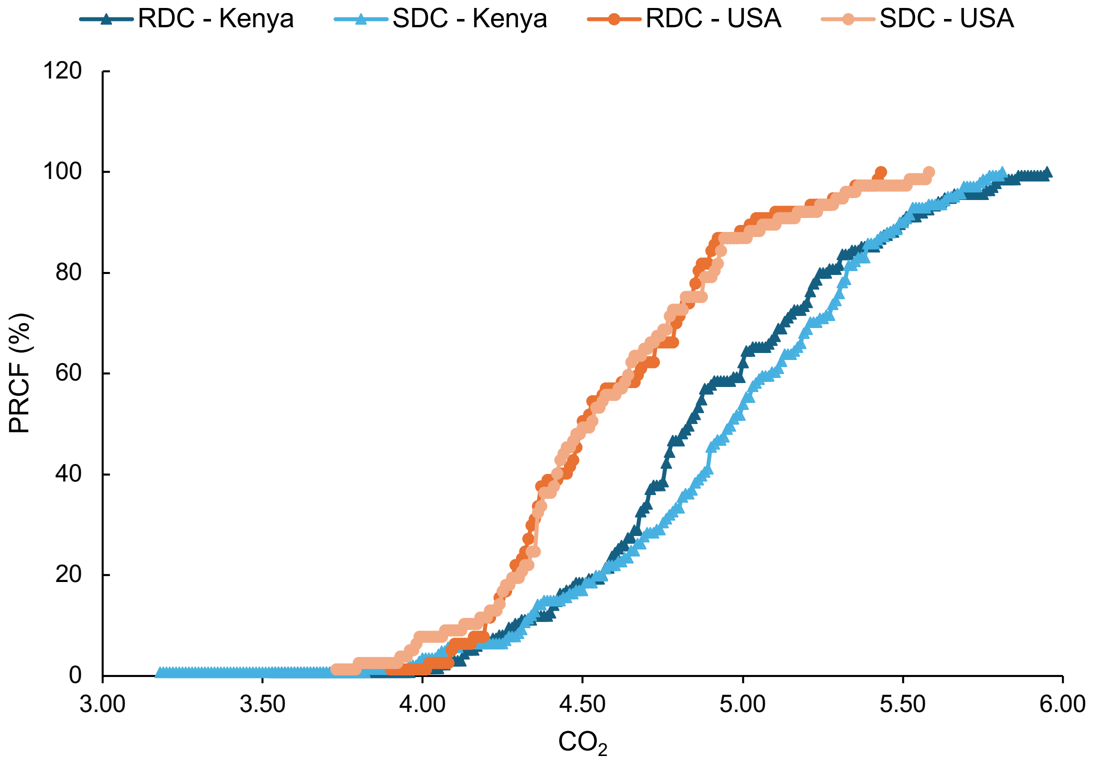

Percent Relative Cumulative Frequency (PRCF) analysis for CO_2 values from **0-50 min**.

Figure 6

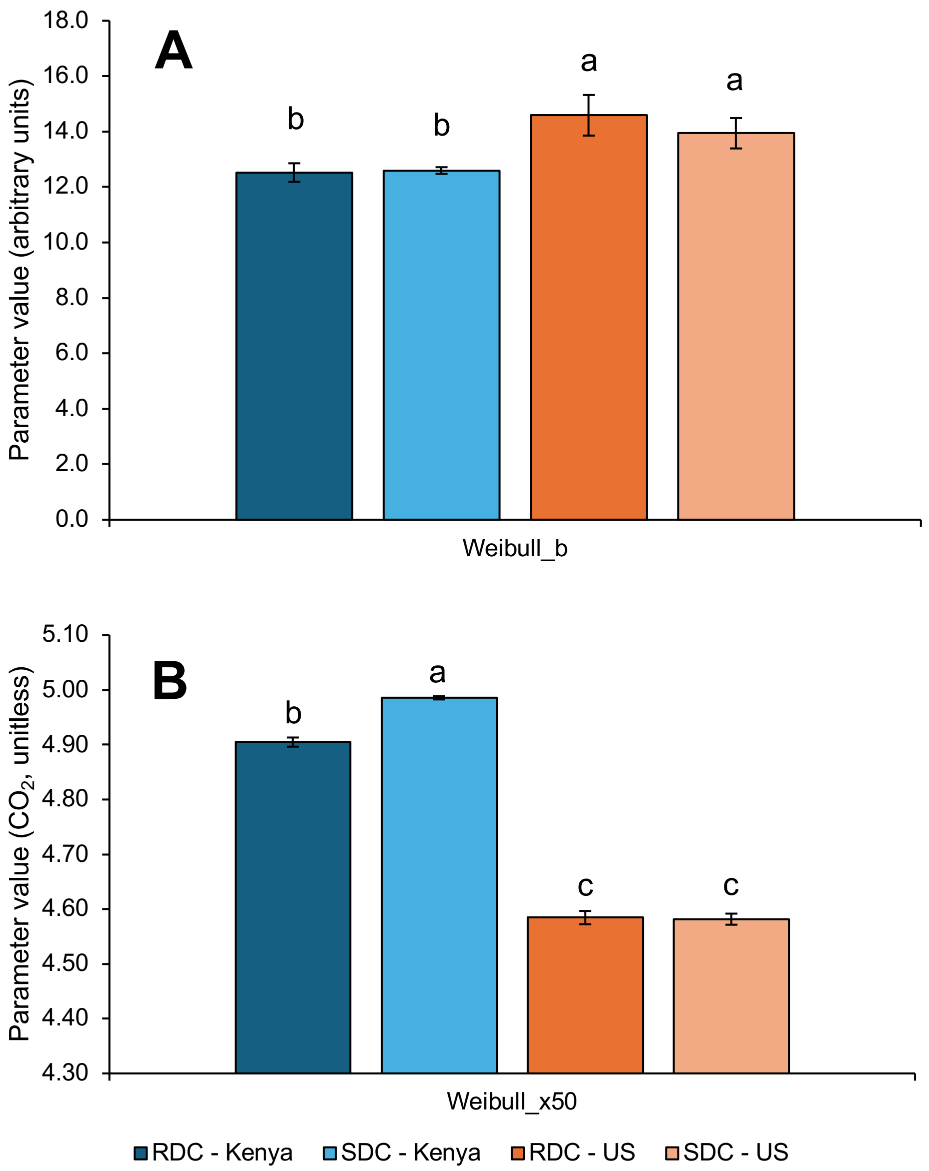

Weibull fit parameter estimates for CO_2 data from **0-50 min**. Error bars represent ±95% confidence intervals (CI).
